# E3 Ligase SCF^βTrCP^-induced DYRK1A Protein Degradation Is Essential for Cell Cycle Progression in HEK293 Cells*

**DOI:** 10.1074/jbc.M116.717553

**Published:** 2016-11-02

**Authors:** Qiang Liu, Yu Tang, Long Chen, Na Liu, Fangfang Lang, Heng Liu, Pin Wang, Xiulian Sun

**Affiliations:** From the ‡ Brain Research Institute,; ¶ Department of Neurology,; ‖ National Key Lab of Otolaryngology, and; ** Department of Hematology, Qilu Hospital of Shandong University, Jinan 250012,; the § Medical Research Center, Shandong Provincial Qianfoshan Hospital, Shandong University, 10766 Jingshi Road, Jinan 250014, and; the ‡‡ Department of Gynecology and Obstetrics, Jinan Central Hospital Affiliated with Shandong University, 105 Jiefang Road, Jinan 250013, China

**Keywords:** Alzheimer's disease, cell cycle, DYRK1A, E3 ubiquitin ligase, protein degradation, SCFβTrCP

## Abstract

DYRK1A, located on the Down syndrome (DS) critical region of chromosome 21, was found to be overexpressed in brains of DS and Alzheimer's disease individuals. DYRK1A was considered to play important roles in the pathogenesis of DS and Alzheimer's disease; however, the degradation mechanism of DYRK1A was still unclear. In this study, we found that DYRK1A was degraded through the ubiquitin-proteasome pathway in HEK293 cells. The N terminus of DYRK1A that was highly unstable in HEK293 cells contributed to proteolysis of DYRK1A. E3 ligase SCF^βTrCP^ mediated ubiquitination and promoted degradation of DYRK1A through an unconserved binding motif (^49^SDQQVSALS^57^) lying in the N terminus. Any Ser-Ala substitution in this motif could decrease the binding between DYRK1A and β-transducin repeat containing protein (βTrCP), resulting in stabilization of DYRK1A. We also found DYRK1A protein was elevated in the G_0_/G_1_ phase and decreased in the S and G_2_/M phase, which was negatively correlated to βTrCP levels in the HEK293 cell cycle. Knockdown of βTrCP caused arrest of the G_0_/G_1_ phase, which could be partly rescued by down-regulation of DYRK1A. Our study uncovered a new regulatory mechanism of DYRK1A degradation by SCF^βTrCP^ in HEK293 cell cycle progression.

## Introduction

DYRK1A, a member of the dual specificity tyrosine phosphorylation-regulated kinase family, located on chromosome 21, was reported to be overexpressed and responsible for nervous defects in patients with Down syndrome (DS)^2^ (1, 2). Several mouse models showed that DYRK1A knock-in could lead to neurodevelopmental delay, motor abnormalities, mental retardation, learning and memory deficits, and reduced neuronal density (3–5), which could also be observed in DS patients (6–8), indicating the important roles of DYRK1A in the development and functions of the nervous system. Recent studies provided evidence that neural functional defects in DS could be due to proliferative impairment of neural progenitor cells. Decreased proliferative activity in the ventricular zone/subventricular zone and dentate gyrus was observed in DS patients as early as the fetal phase (9–11). As one of the key genes implicated in DS, DYRK1A was considered to be involved in the proliferative regulation of NPCs. Studies showed that overexpression of DYRK1A could inhibit proliferation and promote cell cycle exit of NPCs by phosphorylation on several cell cycle regulators, including cyclin D1 (12–14) and p27^Kip^ (14, 15), which was consistent with the proliferative disruption of NPCs in DS. However, haploinsufficency of DYRK1A could lead to impaired longevity of neural stem cells, resulting in reduced self-renewal and reservoir of neural stem cells (15, 16). These studies indicate the importance of maintaining the appropriate protein level of DYRK1A in neural development. Our recent study showed that RE1 silencing transcription factor (REST) regulated DYRK1A gene transcription, and both the overexpression and down-regulation of DYRK1A reduced REST levels, further emphasizing the appropriate protein level of DYRK1A for cellular functions (17).

Nearly all DS individuals showed typical features of AD, such as senile plaques and neurofibrillary tangles in their early 40s, making DS an ideal model for AD research (18). Studies showed that DYRK1A could phosphorylate the amyloid protein precursor on Thr-668 (19) and several sites of Tau (20–22). Phosphorylation by DYRK1A could facilitate cleavage of APP by the β-site APP cleaving enzyme 1 (23) and γ-secretase (23, 24), leading to increased production of amyloid-β. DYRK1A-mediated phosphorylation could disrupt the normal biological effect of Tau and make it much more preferable to aggregate (22). The roles of DYRK1A in AD pathogenesis were still not clear. Nonetheless, histological studies showed that there were significantly more DYRK1A-positive cells in AD brains than normal controls (25). The protein level of DYRK1A was also remarkably up-regulated in the sporadic AD cortex, compared with normal individuals (25). However, the detailed mechanism causing abnormal up-regulation of DYRK1A in AD brains was still unclear. The ubiquitin-proteasome pathway, one of the main routes for protein destruction in eukaryotic cells, has been implicated in maintaining normal functions of nervous systems. Accumulation of ubiquitin conjugates or aggregates has been reported in AD and other neural degenerative diseases (26), implying that ubiquitin-proteasome-mediated protein degradation was impaired during the pathogenesis of neural degeneration. DYRK1A showed strong gene dosage effect, and its transcription and translation were strictly regulated (27). As described above, DYRK1A overexpression in DS patients was due to the extra copy of the DYRK1A gene. However, generally AD patients had normal gene copy number, suggesting that disrupted proteolysis could be responsible for DYRK1A increments in AD patients. Nevertheless, the molecular mechanism of DYRK1A degradation was still unclarified.

In the ubiquitin-proteasome system, ubiquitin was sequentially catalyzed by the ubiquitin-activating enzyme (E1), ubiquitin-conjugating enzyme (E2), and ubiquitin ligase (E3) to be covalently linked to Lys residues of substrates, forming a degradation signal that could be recognized by 26S proteasome (26). SCF^βTrCP^ was the most frequently studied ubiquitin ligase. It could specifically bind to the DpSG*XX*pS motif, where serine residues were phosphorylated, and subsequently mediated by ubiquitination of substrates (28, 29). Ubiquitin cluster covalently connected to substrates functioned as a degradation signal and could be recognized by proteasome for degradation (26). SCF^βTrCP^ exerted its roles in neural development by regulating the stability of the RE1-silencing transcription factor, which was a transcriptional repressor of neural genes in non-neuronal cells. SCF^βTrCP^ promoted neuronal differentiation by targeting REST for degradation in the proteasome (30). In our previous study, we found that dysregulation of DYRK1A could impair REST protein stability, making REST vulnerable for proteolysis (17). However, more studies are needed to uncover whether there is an association between SCF^βTrCP^ and DYRK1A in neural development.

In this study, we found DYRK1A was degraded through the ubiquitin-proteasome pathway in HEK293 cells. Its N terminus rather than the PEST-rich region was responsible for the proteasome proteolysis. SCF^βTrCP^ bound to the ^49^SDQQVSALS^57^ motif lying in the N terminus of DYRK1A, mediating the ubiquitination of DYRK1A. Knockdown of βTrCP up-regulated DYRK1A protein levels and prolonged its half-life. We also observed that DYRK1A protein but not mRNA was elevated in the G_0_/G_1_ phase and decreased during S and G_2_/M phase, negatively correlated with the changes of βTrCP in HEK293 cell cycle. Our study proposed a new regulatory role of DYRK1A proteolysis in HEK293 cell cycle progression.

## Results

### 

#### 

##### DYRK1A Degradation Was Mediated by Ubiquitin-Proteasome System

**FIGURE 1.**
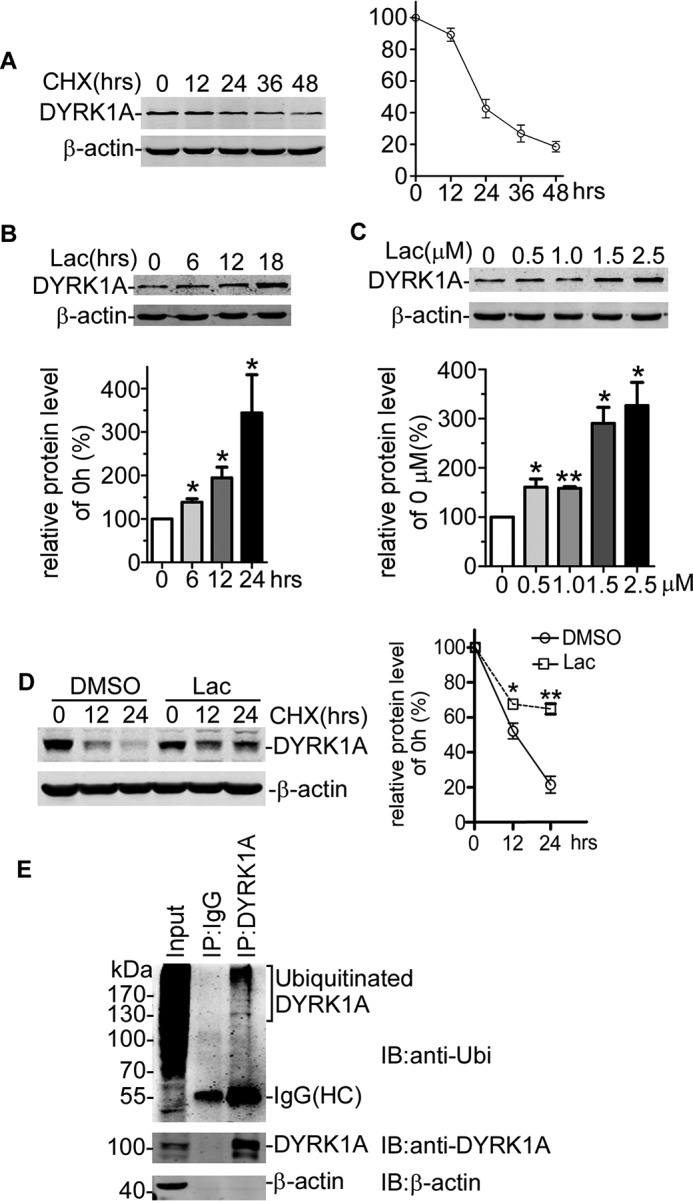
**DYRK1A degradation was mediated by ubiquitin-proteasome system.** *A,* HEK293 cells were transfected with pDYRK1A-MycFLAG, and CHX chase assay was applied to determine the half-life of DYRK1A. FLAG antibody was used to detect exogenous DYRK1A. β-Actin was used as the loading control. *B* and *C,* HEK293 cells were transfected with DYRK1A-expressing construct. Cells were then treated with 2.5 μm Lac for the indicated time or a series of dosages of Lac for 24 h. Cell lysate was analyzed by Western blotting. FLAG antibody was used to detect the protein level of DYRK1A. β-Actin was used as loading control. Values represented means ± S.E. (*n* = 3). *, *p* < 0.05; **, *p* < 0.01. *D,* HEK293 cells were transfected with DYRK1A-expressing vector. Thirty six hours after transfection, cells were treated with 2.0 μm lactacystin for 2 h. After that, CHX assay was performed to detect the degradation of DYRK1A. FLAG antibody was used to tag exogenous DYRK1A. β-Actin was used as internal control. *, *p* < 0.05; **, *p* < 0.01. *E,* HEK293 cells were lysed in 1% Nonidet P-40 lysis buffer. Endogenous DYRK1A protein was concentrated by DYRK1A (7D10) antibody. Ubiquitin antibody was used to detect ubiquitinated DYRK1A. *IB,* immunoblot.

To determine the half-life of DYRK1A, HEK293 cells were pulse-chased with CHX. Cells were harvested every 12 h after CHX treatment, and DYRK1A protein was analyzed by Western blotting. As shown in Fig. 1*A*, DYRK1A protein gradually decreased in a time-dependent manner after the addition of CHX. The half-life of DYRK1A in HEK293 cells was 14.07 h by the following calculation, *Q_t_* = *Q*_0_·(1/2)*^t^*/H, where *Q_t_* indicates the protein level at *t* time; *Q*_0_ indicates the protein level at 0 h; and *H* indicates half-life (Fig. 1*A*).

Ubiquitin-proteasome pathway was responsible for degradation of most proteins in eukaryotic cells. To examine whether the ubiquitin-proteasome pathway was involved in thedegradation of DYRK1A, HEK293 cells were transfected with pDAYRK1A-MycFLAG and treated with the proteasome inhibitor lactacystin. Western blotting results clearly showed that treatment with lactacystin significantly increased DYRK1A protein level in a time-dependent (Fig. 1*B*) and dosage-dependent manner (Fig. 1*C*). Lactacystin treatment also markedly decreased the degradation of DYRK1A in CHX chase assay (Fig. 1*D*). These data demonstrated that ubiquitin-proteasome participated in the degradation of DYRK1A.

Because ubiquitination was the essential step for ubiquitin-proteasome pathway-mediated protein degradation, we next investigated whether DYRK1A could be ubiquitinated using co-IP assay. Endogenous DYRK1A was pulled down with DYRK1A monoclonal antibody, and ubiquitinated proteins were detected by ubiquitin antibody. Western blotting exhibited a smeared band, the typical feature of ubiquitination (Fig. 1*E*), implying that DYRK1A could be ubiquitinated in HEK293 cells. Taken together, our results revealed that DYRK1A was ubiquitinated and degraded by the ubiquitin-proteasome system.

##### Down-regulation of DYRK1A Rescued Cell Cycle Arrest at G_0_/G_1_ Phase

**FIGURE 2.**
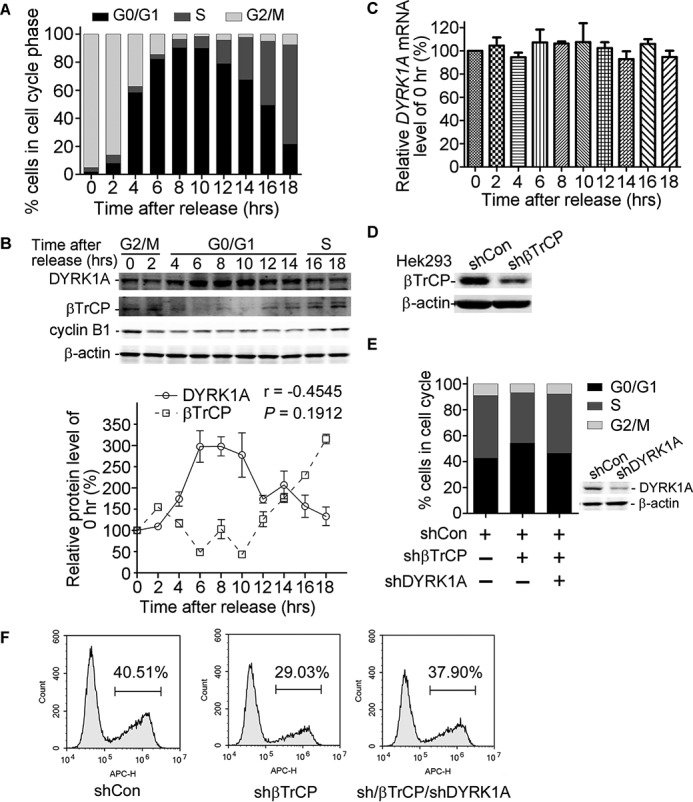
**Expression profile of DYRK1A in cell cycle and down-regulation of DYRK1A rescued cell cycle arrest at G_0_/G_1_ phase.** HEK293 cells were treated with nocodazole with a final concentration of 150 ng/ml for 12 h. Then fresh medium was added, and cells were harvested every 2 h. Then FACS (*A*), Western blotting (*B*), and real time qRT-PCR (*C*) were performed to detect cell cycle, DYRK1A and βTrCP protein, and DYRK1A mRNA, respectively. β-Actin was used as internal control for both Western blotting and real time qRT-PCR. Values represented means ± S.E. (*n* = 3). Pearson's correlation test was used for correlation analysis between DYRK1A and βTrCP protein levels (*r* = −0.5655, *p* = 0.0442). *D,* endogenous βTrCP in HEK293 stably expressing βTrCP shRNAs was determined by Western blotting. β-Actin was used as loading control. *E,* stably transformed HEK293 cell line was transfected with DYRK1A shRNA expressing or control vectors. Thirty six hours after transfection, cells were stained with propidium iodide and subjected for FACS analysis. *F,* βTrCP stably transfected HEK293 cell line was transfected with DYRK1A shRNA construct. Thirty six hours after transfection, cells were replaced with fresh medium containing 10 μm EdU. After 10 h of incubation, cells were collected and stained with Apollo 643 reagent. Positive-staining cells were counted on a FACS instruments.

In cell cycle progression, E3 ligase SCF^βTrCP^ showed the highest activity in S phase, and βTrCP was specifically attenuated in the G_0_/G_1_ phase and increased in the S phase, implying the important roles of SCF^βTrCP^ during G_0_/G_1_-S phase transition. DYRK1A had been reported to be involved in regulating cell proliferation. We previously showed that overexpression of DYRK1A could cause proliferative inhibition and cell cycle arrest at the G_0_/G_1_ phase, indicating that down-regulation of DYRK1A could be necessary for G_0_/G_1_-S phase transition (31). To test this possibility, we synchronized HEK293 cells by nocodazole. After 12 h of treatment with nocodazole, more than 90% of cells were arrested at G_2_/M phase (Fig. 2*A*). Cell cycle was recovered with fresh medium. We found DYRK1A protein showed the highest expression level at the G_0_/G_1_ phase and was comparatively reduced at the S and G_2_/M phase (Fig. 2*B*). Interestingly, βTrCP protein level was negatively correlated to DYRK1A, exhibiting an inverse profile in cell cycle (Pearson *r* = −0.5655, *p* = 0.0442, Fig. 2*B*). The down-regulation of DYRK1A during G_0_/G_1_-S phase transition could be due to enhanced degradation, as the mRNA level of DYRK1A did not change significantly in cell cycle (Fig. 2*C*). Then we established a HEK293 cell line stably expressing βTrCP shRNAs (Fig. 2*D*), and we evaluated the cell cycle of this cell line. FACS results showed that βTrCP shRNAs reduced the ratio of cells in S phase by 9.12% and increased the ratio of cells in G_0_/G_1_ phase by 11.53%. When transfected with DYRK1A interfering constructs, the cell cycle arrest induced by βTrCP knockdown was partly rescued (Fig. 2*E*). To further confirm our results, we performed an EdU staining assay. Flow cytometry analysis showed that knockdown of βTrCP markedly decreased positive-staining cells from 40.51 to 29.03%, which could be rescued by DYRK1A shRNA transfection (37.90%) (Fig. 2*F*). Our studies suggested that DYRK1A was inversely regulated by E3 ligase SCF^βTrCP^ and played important roles in cell cycle progression.

##### DYRK1A Degradation Was Mediated by E3 Ligase SCF^βTrCP^

**FIGURE 3.**
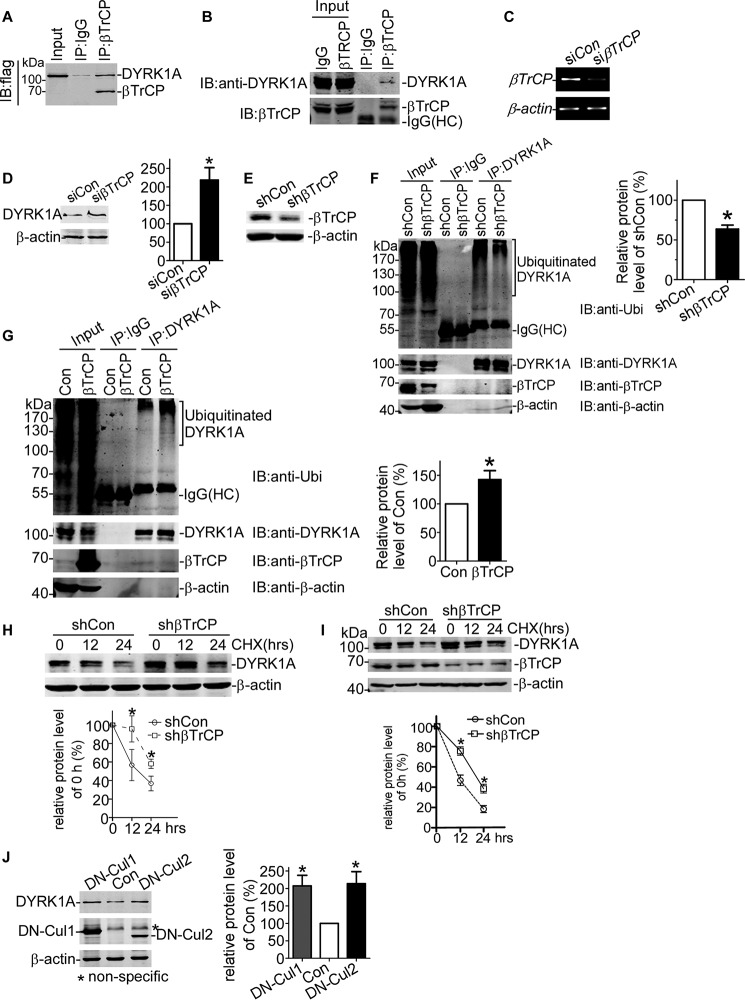
**E3 ligase SCFβTrCP promoted DYRK1A degradation by accelerating its ubiquitination.** *A,* HEK293 cells were co-transfected with DYRK1A and βTrCP-expressing constructs. Co-IP assay was performed to determine the interaction between DYRK1A and βTrCP. βTrCP antibody was applied in the pulldown step. FLAG antibody was used for signal detection. *B,* co-IP assay was used to detect the interaction between intrinsic endogenous DYRK1A and βTrCP. βTrCP and DYRK1A antibodies were used as the IP and immunoblot (*IB*) antibody, respectively. IgG (rabbit) was used as the negative control of βTrCP antibody. *C,* HEK293 cells were transfected with βTrCP siRNAs (*si*β*TrCP*) or negative control (*siCon*). Total RNA was separated by TRIzol reagent. The mRNA level of β*TrCP* was evaluated by qRT-PCR. *D,* HEK293 cells were co-transfected with DYRK1A-expressing plasmid and βTrCP or control siRNAs. DYRK1A protein level was detected by Western blotting using FLAG antibody. β-Actin was used as internal control. Values represented means ± S.E. (*n* = 3). *, *p* < 0.05. *E,* protein level of DYRK1A in βTrCP shRNA lentivirus-infected HEK293 cells was determined by Western blotting. β-Actin was used as internal control. *F,* co-IP assay was performed to detect the ubiquitinated DYRK1A in cells stably transfected with of sh-βTrCP. IgG was used as negative control of DRK1A (7D10) antibody. The ubiquitination was normalized to pulled down DYRK1A. Values represented means ± S.E. (*n* = 3). *, *p* < 0.05. *G,* ubiquitinated DYRK1A was estimated by co-IP assay in cells transfected with βTrCP expression vector. DYRK1A (7D10) antibody was used as pulldown antibody, and IgG was used as negative control. The ubiquitination was normalized to pull down DYRK1A. Values represented means ± S.E. (*n* = 3). *, *p* < 0.05. *H,* degradation rate of DYRK1A in shCon and shβTrCP lentivirus-transformed HEK293 cells was determined using CHX chase assay. DYRK1A was detected by FLAG antibody. β-Actin was used as internal control. Values represented means ± S.E. (*n* = 3). *, *p* < 0.05. *I,* degradation of endogenous DYRK1A was determined by CHX chase assay. DYRK1A and βTrCP antibody were used to detect endogenous DYRK1A and βTrCP, respectively. β-Actin was used as internal control. Values represented means ± S.E. (*n* = 3). *, *p* < 0.05. *J,* DYRK1A was co-transfected with Cullin1 or Cullin2 dominant negative mutants expressing constructs. Protein level of DYRK1A was evaluated by Western blotting using FLAG antibody. β-Actin was used as internal control. Values represented means ± S.E. from three independent experiments. *, *p* < 0.05. *IB,* immunoblot.

As the key component of the SCF^βTrCP^ complex, F-box protein βTrCP directly bound to target proteins independent of the SCF complex, promoting ubiquitination of target proteins (26). To investigate whether SCF^βTrCP^ was involved in DYRK1A degradation, we first applied co-IP assay to detect the interaction between βTrCP and DYRK1A. Co-IP results showed that both exogenous βTrCP and DYRK1A could be efficiently precipitated by βTrCP antibody (Fig. 3*A*). To better validate the interaction between βTrCP and DYRK1A, we performed co-IP using HEK293 cells without any treatment. This experiment also revealed that endogenous βTrCP and DYRK1A could be precipitated simultaneously (Fig. 3*B*). These results indicated that βTrCP interacted with DYRK1A.

To demonstrate whether SCF^βTrCP^ was involved in DYRK1A protein degradation, we interrupted SCF^βTrCP^ by knocking down βTrCP expression with siRNAs (Fig. 3*C*). βTrCP siRNA significantly increased the DYRK1A protein level to 218.51 ± 33.61%, compared with control (Fig. 3*D*). To further confirm the roles of βTrCP in the degradation of DYRK1A, we assessed the ubiquitination status of DYRK1A when βTrCP was knocked down or overexpressed. We infected HEK293 cells with βTrCP shRNA-expressing lentiviral particles (Fig. 3*E*). Our results showed βTrCP down-regulation led to a significant decrease of ubiquitinated endogenous DYRK1A levels to 61.72 ± 8.74% (Fig. 3*F*), whereas overexpression of βTrCP remarkably promoted the ubiquitination of endogenous DYRK1A to 142.73 ± 26.98% (Fig. 3*G*). To validate whether DYRK1A up-regulation upon βTrCP interference was due to impaired proteolysis, we determined the DYRK1A turnover rate in HEK293 cells with the βTrCP knocked out. CHX chase assay showed that both the exogenous and endogenous DYRK1A proteins (Fig. 3, *H* and *I*, respectively) were markedly stabilized in shβTrCP stably transfected HEK293 cells, implying an important role of βTrCP in the proteolytic process of DYRK1A.

The SCF^βTrCP^ E3 ligase complex was mainly composed of four core components as follows: Skp1; F-box protein (βTrCP); cullin; and Rbx1. βTrCP specifically interacted with target proteins, whereas cullin acted as the structural scaffold linking the N terminus of Skp1 to the C terminus of Rbx1 (28). To disrupt the normal function of SCF^βTrCP^ complex, we constructed dominant negative mutants of cullin, in which only the N-terminal 1–452-aa residues of Cullin1 or 20–427-aa residues of Cullin2 were expressed (DN-Cullin1 and DN-Cullin2). These dominant negative forms of cullin, serving as the decoy partner, could assemble a complex with Skp1 and F-box protein (βTrCP), while losing the ability to bind RBX1 and E2 enzyme, leading to failure of ubiquitination. When overexpressed with DN-Cullin1 or DN-Cullin2, the protein level of DYRK1A was increased to 207.44 ± 30.17 and 213.73 ± 34.50%, compared with control vector, respectively (Fig. 3*J*). Our studies provided solid evidence that E3 ligase SCF^βTrCP^ was responsible for DYRK1A proteolysis.

##### DYRK1A Degradation Was Mediated by Its N Terminus

**FIGURE 4.**
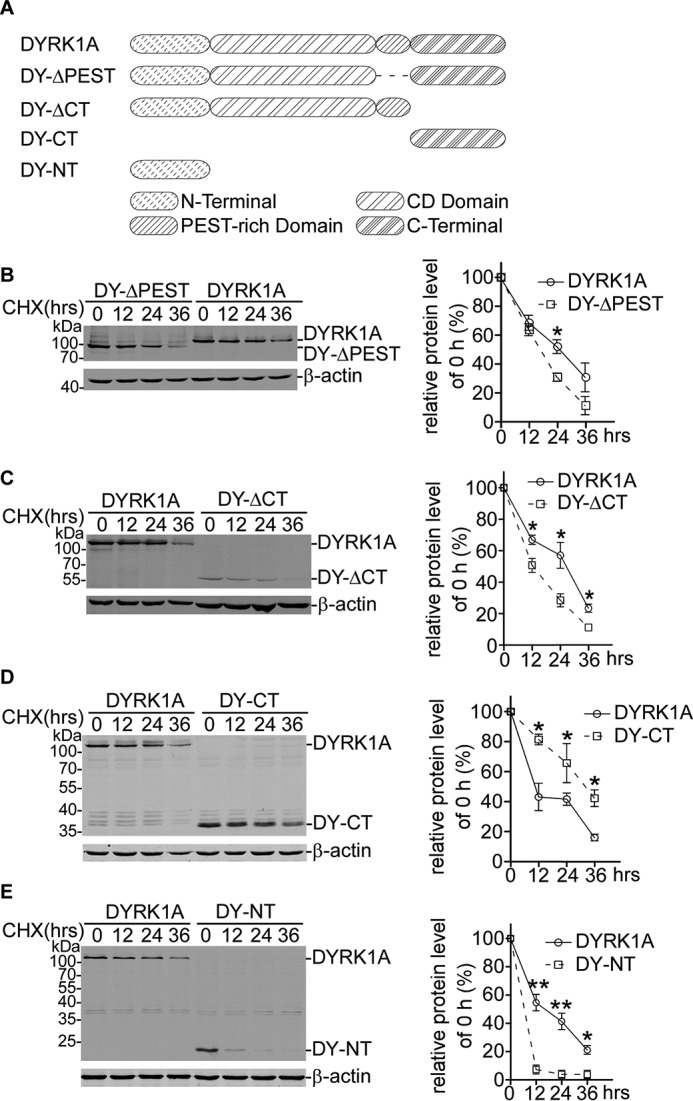
**DYRK1A degradation was mediated by its N terminus rather than the PEST-rich region.** *A,* schematic structure of DYRK1A protein and truncation mutation strategy. Expressing plasmids of wild type DYRK1A or truncation mutants DY-ΔPEST (*B*), DY-ΔCT (*C*), DY-CT (*D*), and DY-NT (*E*) were transfected into HEK293 cells. CHX chase assay was performed to determine the degradation dynamics. Values represented means ± S.E. (*n* = 3). *, *p* < 0.05; **, *p* < 0.01.

DYRK1A protein was composed of the following four domains: N terminus (NT, 1–149 aa); the kinase catalytic domain (150–477 aa); the PEST-rich region (PEST, 478–516 aa); and the C-terminal (CT, 517–754 aa) (Fig. 4*A*) (32). The PEST-rich region was a peptide composed with proline (P), glutamic acid (E), serine (S), and threonine (T) residues, commonly acting as protein degradation signal (33).

Because DYRK1A possessed a PEST-rich region comprising 478–516-aa residues, it is predicted that DYRK1A could be degraded rapidly with a short half-life in cells. We determined whether the half-life of DYRK1A was dramatically changed when the PEST-rich region was truncated (Fig. 4*A*). CHX chase assay showed that deletion of the PEST-rich region did not significantly alter the degradation rate, compared with wild type DYRK1A (Fig. 4*B*), indicating other domains of DYRK1A could be responsible for DYRK1A proteolysis. For this purpose, a series of DYRK1A truncation mutants were constructed (Fig. 4*A*), and the relevant degradation rates were assessed by CHX chase assay. Compared with wild type DYRK1A, the degradation rate of DY-ΔCT was not significantly changed (Fig. 4*C*), whereas the degradation rate of DY-CT was notably prolonged (Fig. 4*D*), suggesting the C terminus did not play a significant role in DYRK1A degradation. Noticeably, we found that the N terminus of DYRK1A (DY-NT) was highly unstable and could be rapidly degraded in HEK293 cells (Fig. 4*E*), compared with other domains of DYRK1A. These results suggested the N terminus could play a vital role in regulating DYRK1A degradation.

##### SCF^βTrCP^ Promoted DYRK1A Degradation via Its N-terminal 34–71 Amino Acids

**FIGURE 5.**
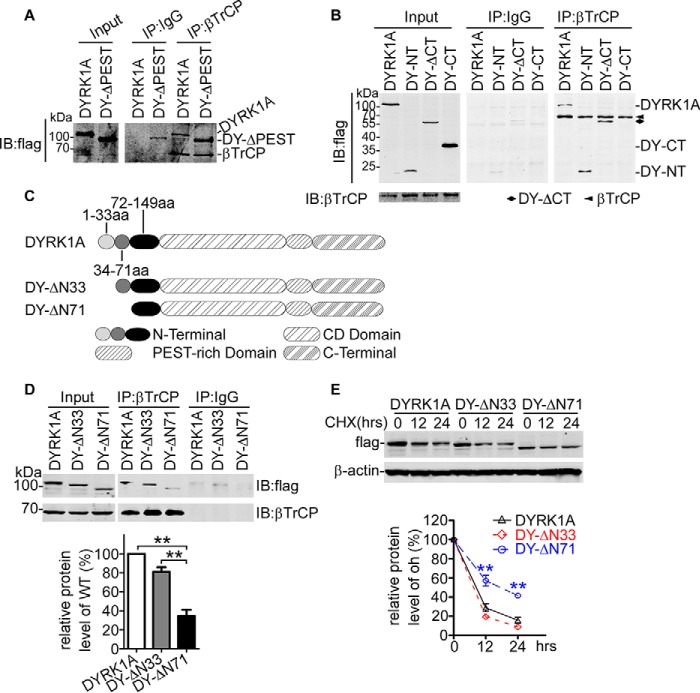
**SCF^βTrCP^ bound to DYRK1A N-terminal 71-aa residues and promoted its degradation.** *A,* βTrCP-expressing vector and DYRK1A wild type plasmid DY-WT or PEST-truncated mutant DY-ΔPEST were co-transfected into HEK293 cells. Two days later, cells were harvested and lysed in 1% Nonidet P-40 lysis buffer. Co-IP assay was performed using βTrCP antibody and protein A/G-agarose beads. IgG (rabbit) was used as negative control of IP antibody. FLAG antibody was applied for Western blotting analysis. *B,* HEK293 cells were co-transfected withβTrCP and DYRK1A-, DY-NT-, DY-ΔCT-, or DY-CT-expressing construct. In co-IP assay, βTrCP and FLAG antibody were used as IP and IB antibody, respectively. IgG (rabbit) was used as negative control of βTrCP antibody. *C,* stable HEK293 cells were transfected with the DYRK1A N terminus expressing construct. CHX chase assay was performed to determine the degradation of the DYRK1A N terminus. FLAG and βTrCP antibody were used to detect DYRK1A N terminus and βTrCP. β-Actin was used as loading control. Values represented means ± S.E. (*n* = 3). *, *p* < 0.05; **, *p* < 0.01. *D,* schematic structure of DYRK1A protein and truncation mutation in the N terminus. *E,* βTrCP and DYRK1A-, DY-ΔN33-, or DY-ΔN71-expressing plasmids were co-transfected into HEK293 cells. βTrCP was pulled down by βTrCP antibody, and IgG (rabbit) served as negative control. FLAG antibody was used to detect βTrCP-interacted DYRK1A, DY-ΔN33, or DY-ΔN71. Quantification was calculated from three independent experiments, normalized to βTrCP pulled down. Values represented means ± S.E. **, *p* < 0.01. Cells were subjected to CHX chase assay. Protein level of DYRK1A, DY-ΔN33, and DY-ΔN71 was determined by FLAG antibody. β-Actin was used as internal control. Values represented means ± S.E. (*n* = 3). **, *p* < 0.01. *IB,* immunoblot.

Considering the importance of E3 ligase SCF^βTrCP^ and N terminus in the degradation of DYRK1A, we proposed that SCF^βTrCP^ could directly bind to the N terminus of DYRK1A. To confirm this, we carried out co-IP assay between βTrCP and a series of DYRK1A truncation mutants (Fig. 4*A*). Results in Fig. 5, *A* and *B,* show that only DYRK1A truncation mutants containing the N terminus could be co-immunoprecipitated with βTrCP, implying that the SCF^βTrCP^ complex specifically bound to the N terminus of DYRK1A. To further identify the exact residues to which βTrCP bound, we constructed another two truncation mutants, which lacked the N-terminal 33 aa (DY-ΔN33) or 71 aa (DY-ΔN71) residues (Fig. 5*C*). The co-IP results showed that interaction between DY-ΔN33 and βTrCP was negligibly affected (Fig. 5*D*, *5th lane versus 4th lane*), but the DY-ΔN71 band co-immunoprecipitated with βTrCP was markedly reduced (Fig. 5*D*, *6th lane versus 4th lane*), suggesting that the region of 34–71 aa contained the degradation degron. To further verify this degradation degron, the CHX assay was performed in HEK293 cells transfected with the two deletion constructs. The CHX chase assay demonstrated that the degradation rate of DY-ΔN33 was similar to that of wild type DYRK1A, whereas DY-NΔ71 degradation was noticeably impaired, revealed by slower decrement after CHX addition (Fig. 5*E*). Collectively, our results suggested the binding site of βTrCP on the DYRK1A protein lying in the N-terminal 33–71-aa residues.

##### SCF^βTrCP^ Bound to an Unconserved Motif in the DYRK1A N Terminus

**FIGURE 6.**
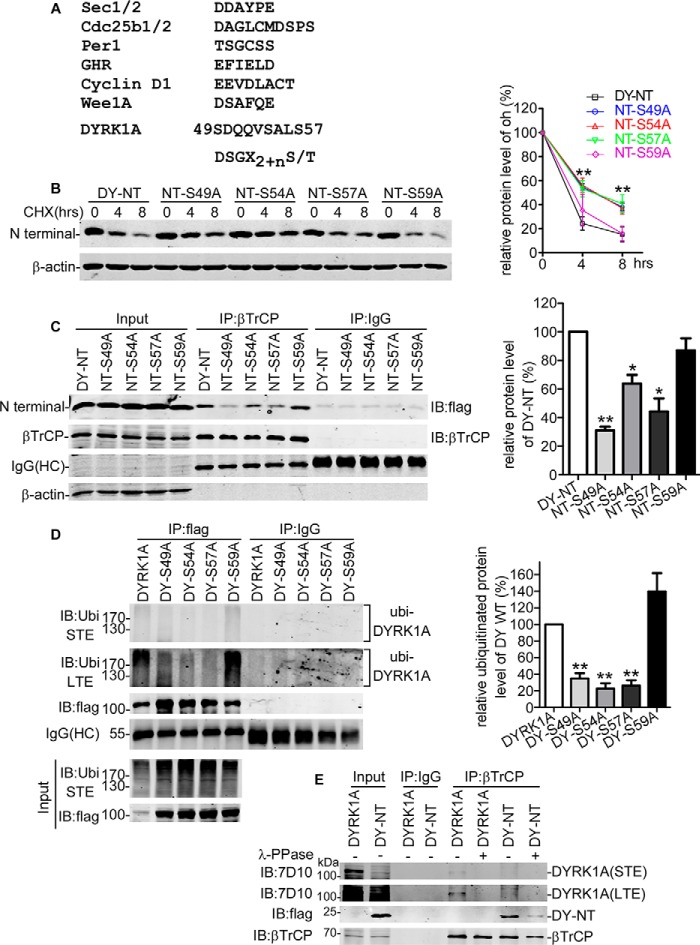
**SCF^βTrCP^ bound to an unconserved motif of the DYRK1A N terminus.** *A,* lists of unconserved SCF^βTrCP^ substrate sequences and predicted binding motif in DYRK1A. *B,* DY-NT or S49A, S54A, S57A, and S59A mutants were transfected into HEK293 cells. Thirty six hours later, CHX chase was performed to evaluate the degradation rate. FLAG antibody was applied for Western blotting analysis. β-Actin was used as loading control. The quantification was calculated from three independent experiments. Values represented means ± S.E. **, *p* < 0.01. *C,* expressing constructs of βTrCP and DY-NT or S49A, S54A, S57A, and S59A mutants were co-transfected into HEK293 cells. Forty eight hours after transfection, co-IP assay were applied utilizing βTrCP antibody. IgG (rabbit) was used as negative control. FLAG antibody, βTrCP, and β-actin antibody were used to detect Western blotting signal. The band intensity of DYRK1A N-terminal wild type and mutants was normalized to pull down βTrCP. Values represented means ± S.E. (*n* = 3). *, *p* < 0.01; **, *p* < 0.01. *D,* ubiquitin and DYRK1A full-length wild type or point mutants DY-S49A-, DY-S54A-, DY-S57A-, or DY-S59A-expressing plasmids were co-transfected into HEK293 cells. Co-IP was executed using FLAG antibody 48 h later. Precipitated proteins were detected by ubiquitin (*Ubi*) and FLAG antibody. Values represented means ± S.E. (*n* = 3). **, *p* < 0.01. *STE*, short time exposure. *LTE*, long time exposure. *E,* βTrCP and DYRK1A or its N-terminal expressing constructs were co-transfected into HEK293 cells. 48 h later, cells were collected and lysed in 1% Nonidet P-40 lysis buffer. Protein samples were dephosphorylated by incubating with λ-PPase for 1 h at 30 °C and subsequently subjected to co-IP assay using βTrCP antibody. IgG (rabbit) was used as negative control. Western blotting signals were detected by DYRK1A (7D10), FLAG, and βTrCP antibody. *STE,* short time exposure. *LTE,* long time exposure.

SCF^βTrCP^ was the substrate-specific E3 ligase that directly interacted with a conserved binding motif DSGX2+*n*(S/T), where serines were phosphorylated by particular kinases (34). Despite the substrate preference, many studies also showed that SCF^βTrCP^ complex could also recognize proteins lacking the perfect DSGX2+*n*S(T) motif and promote ubiquitination (35–40). However, most of these substrates harbored at least one variant of the DSGX2+*n*(S/T) motif (Fig. 6*A*). Normally, substitutions between phosphorylated residues and aspartic or glutamic acid existed in these variants (Fig. 6*A*), forming a functional degron. Comparing DYRK1A N terminus with DSG*XX*S and its variants, we identified a similar sequence ^49^SDQQVSALS^57^ (Fig. 6*A*).

To investigate the role of SDQQVSALS sequence in the degradation of DYRK1A, we mutated the Ser-49, Ser-54, Ser-57, and Ser-59 residue to alanine in the DY-NT construct. First, we utilized CHX chase assay to determine the degradation rate of wild type DYRK1A N terminus and NT-S49A, NT-S54A, NT-S57A, and NT-S59A mutants. Western blotting results showed that N-terminal proteolysis was significantly slowed down by single amino acid substitution on Ser-49, Ser-54, or Ser-57 but not Ser-59 (Fig. 6*B*), suggesting ^49^SDQQVSALS^57^ motif could be responsible for DYRK1A degradation. To further confirm this, we examined the interactions of DYRK1A N-terminal mutants with βTrCP as described previously. The N terminus of DYRK1A could efficiently interact with βTrCP, and the NT-S59A mutant showed similar binding ability (Fig. 6*C*). However, S49A, S54A, or S57A mutation significantly reduced the interaction between the DYRK1A N terminus and βTrCP (Fig. 6*C*).

In the process of ubiquitination, E3 ligase recruited E2 ligase and substrate and catalyzed the transfer of ubiquitin from E2 ligase to substrate. We speculated that ubiquitination of DYRK1A could be disrupted if its interaction with SCF^βTrCP^ was impaired. To test this, we pulled down wild type DYRK1A as well as DYRK1A S49A, S54A, S57A, and S59A mutants to assess the comparative ubiquitination level. We found that substitution of amino acid residues on Ser-49, Ser-54, and Ser-57 markedly decreased the relative ubiquitination level (Fig. 6*D*, *2nd* to *4th lanes* compared with *1st lane*). However, DY-S59A mutant failed to exhibit significant alteration on the ubiquitination status (Fig. 6*D*). To determine the effect of phosphorylation in the interaction between βTrCP and DYRK1A or its N terminus, λ-PPase was used to remove the phosphate group from Ser or Thr residues before anti-βTrCP immunoprecipitation. The co-IP assay showed that the binding between βTrCP and DYRK1A or its N terminus was significantly impaired after treatment with λ-PPase (Fig. 6*E*). These data revealed that ^49^SDQQVSALS^57^ as the degradation degron was responsible for DYRK1A ubiquitination and degradation.

## Discussion

In this study, we found that DYRK1A was subjected to degradation in proteasome mediated by E3 ligase SCF^βTrCP^ in HEK293 cells. We identified ^49^SDQQVSALS^57^ as the binding motif of βTrCP, which was located in the N-terminal region but not the PEST-rich region of DYRK1A. The ubiquitin-proteasome pathway was responsible for proteolysis of more than 80% proteins in the eukaryote, playing diverse roles in regulation of proliferation, apoptosis, tumor genesis, and neural development (41). Disruption of the ubiquitin-proteasome pathway played a pivotal role in several neurodegenerative diseases. Complex of ubiquitin, proteasome, and certain proteins was found in neurofibrillary tangles of AD, Lewy bodies (LBs) of Parkinson's disease, LBs in LB dementia, Bunina bodies in amyotrophic lateral sclerosis, and nuclear inclusions of Huntington's disease, spinocerebellar ataxias, and Kennedy's syndrome (42). DYRK1A was elevated in DS and AD patients (2, 25); however, the molecular mechanism was unknown. The study here implied that the disruption of ubiquitin proteosome pathway may contribute to the elevation of DYRK1A in AD patients.

Our study and the recent studies from others proposed that proliferative repression caused by DYRK1A overexpression could be due to cell cycle arrest at G_0_/G_1_ phase (14, 31, 43, 44). Consistent with this, we found that DYRK1A protein level was specifically reduced during the transition from G_0_/G_1_ to S phase in HEK293 cells. Overexpression of DYRK1A could disrupt HEK293 cell cycle progression. Previous work showed that SCF^βTrCP^ actively participated in the regulation of the cell cycle, promoting G_0_/G_1_ to S transition by directing degradation of PFKFB (45) and BORA1 (46). More importantly, βTrCP seemed to be up-regulated in the S phase and to promote G_0_/G_1_ to S phase transition (45, 47, 48). Our results showed the negative correlation in the cell cycle progression between DYRK1A and βTrCP. DYRK1A down-regulation partially rescued G_0_/G_1_ arrest caused by βTrCP knockdown, suggesting that E3 ligase SCF^βTrCP^ was affected on HEK293 cell cycle progression partly through its regulation of DYRK1A degradation. The study used HEK293 cells to study the cell cycle progression, which is a cancer cell line and may not reflect the cell cycle profile of normal cells.

Recent studies in human fetuses and mouse models with DS (9–11) (9, 49, 50) revealed reduced cell proliferation of ventricular and subventricular zones and dentate gyrus, the two neurogenic regions where NPCs mainly existed, suggesting the impaired NPC reservoir could effectively contribute to the pathology of DS. Studies by Hammerle *et al.* (15), Yabut *et al.* (12), and Park *et al.* (44) showed that overexpression of DYRK1A could induce cell cycle exit and proliferative inhibition of NPCs (12, 15, 44), and the proliferative suppression induced by DYRK1A overexpression could be markedly rescued by specific repression of DYRK1A activity by siRNAs, dominant negative inhibitor, or small molecules (15, 19, 51, 52), directly demonstrating the regulatory effects of DYRK1A in NPC self-renewal. The abnormal expression of DYRK1A in DS and AD brains may lead to the incompetence in NPC self-renewal, which may subsequently be attributed to neurodegeneration.

## Materials and Methods

### 

#### 

##### Cell Culture

HEK293 and HEK293T cells were cultured in high glucose Dulbecco's modified Eagle's medium (Gibco) supplemented with 10% fetal bovine serum (Gibco) at 37 °C and 5.0% CO_2_ atmosphere. Before the day of transfection, cells were seeded at 60–70% confluence. Transfection was carried out using Lipofectamine 2000 reagent (Invitrogen), according to the manufacturer's protocols. Lactacystin (Lac) (Sigma) or CHX (Sigma) was added to medium 24 or 36 h after transfection, respectively. For synchronization, cells were treated with 150 ng/ml nocodazole (Sigma) for 12 h and then cultured in fresh medium.

To establish a stable transfection cell line with βTrCP knocked down, HEK293 cells were plated in a 6-well plate with a density of 50,000/well. On the 2nd day, cells were supplemented with shβTrCP lentiviral or control particles. After a 24-h incubation, the medium containing lentiviral particles was removed and replaced with fresh medium. Three days after infection, puromycin (Sigma) was added with a final concentration of 1 μg/ml for 2 weeks to the cell culture for screening, which was sustained for 2 weeks. After screening, the stable transfection cell lines were maintained with puromycin at a low concentration of 0.1 μg/ml.

For transfection, cells are plated into culture dishes at a density of 50,000/cm^2^. The next day, when the cells reach the confluency of 70%, cells were transfected with plasmids or siRNAs utilizing Lipofectamine 2000 reagent (Invitrogen), according to the manufacturer's instructions.

##### Western Blotting and Antibodies

To perform Western blotting analysis, cells were harvested and washed in ice-cold PBS twice, and then lysed by ultrasonication in 0.1% SDS-RIPA lysis buffer (Beyotime Institute of Biotechnology, Haimen, China) in the presence of protease inhibitor mixture (Roche Applied Science). Whole-cell extracts were quantified using the DC protein assay kit (Bio-Rad) and separated by 10% glycine SDS-PAGE. Proteins were transferred onto nitrocellulose membrane under 100 V for 2 h. Nitrocellulose membranes were blocked in 5% bovine serum albumin in TBS-T for 2 h and then incubated in primary antibody dilutions (TBS-T with 1% BSA) overnight. The next day, after washing three times in TBS-T for 5 min, membranes were incubated in fluorescence-conjugated secondary antibody for 30 min at room temperature. Signals were detected on LI-COR Odyssey Infrared Imaging System (LI-COR Biosciences, Lincoln, NE). PageRuler Prestained Protein Ladder was purchased from Fermentas (Vilnius, Lithuania). Primary antibodies used in this study were as follows: FLAG antibody (rabbit) (Sigma); FLAG antibody (Sigma); DYRK1A antibody (7D10) (Sigma); βTrCP antibody (D13F10) (CST, Danvers, MA); cyclin B1 antibody (V152) (CST, Danvers, MA). Secondary antibodies IRDye 680 goat anti-rabbit IgG and IRDye 800CW goat anti-mouse IgG were both purchased from LI-COR Biosciences.

##### Cycloheximide Chase Assay

One day before transfection, HEK293 cells were seeded in 6-well plates. The next day, cells were transfected using Lipofectamine 2000 reagent (Invitrogen). Thirty six hours after transfection, cells were treated with 150 μg/ml CHX and harvested every 4 or 12 h for Western blotting analysis. Half-life of protein was calculated using *Q_t_* = *Q*_0_·(1/2)*^t/H^* (where *Q_t_* is protein level at *t* time; *Q*_0_ is protein level at 0 h; and *H* is half-life).

##### Co-IP Assay

For co-IP assay, cells were harvested and lysed in 1% Nonidet P-40 lysis buffer (1% Nonidet P-40, 50 mm Tris-base, 150 mm NaCl, pH 7.4) containing protease inhibitor mixture (Roche Applied Science). Then Sepharose CL-4B (Sigma) with a 10% total lysate volume was added and then shaken gently for 1 h on ice. We centrifuged the lysate bead mixture at 15,000 rpm at 4 °C for 10 min and carefully remove the supernatant into a new 1.5-ml tube. Twenty microliters of supernatant were isolated and used as input. The target protein was immunoprecipitated by shaking overnight at 4 °C with primary antibodies and protein A/G-agarose beads (Santa Cruz Biotechnology, Santa Cruz, CA). Mouse or rabbit IgG (Beyotime Institute of Biotechnology, Haimen, China) was used as a negative control. The next day, agarose beads were aggregated by centrifugation at 3000 rpm. The pellet was washed with 1% Nonidet P-40 lysis buffer once and ice-cold PBS twice. Then the samples were resuspended in 1× loading buffer (Beyotime Institute of Biotechnology, Haimen, China) diluted with 0.1% SDS-RIPA lysis buffer, denatured on a metal bath at 95 °C for 5 min, and analyzed on 10% glycine SDS-PAGE.

##### Cell Cycle Analysis

Cell cycle was analyzed by flow cytometry. Cells were harvested and washed twice in PBS and then fixed in 75% alcohol overnight at 4 °C. The next day cells were washed twice with cold PBS and resuspended in 1 ml of PBS with 40 mg of propidium iodide and 100 mg of RNase A (Sigma). Then the cells were incubated at 37 °C for 30 min. Samples were then analyzed on a FACS machine (Beckman, CA) with 20,000 counted.

##### Production of Lentivirus

HEK293T cell was plated onto a 10-cm dish 1 day before transfection, reaching 80% confluency. The next day, cells were transfected with expressing plasmid, packaging plasmid, and envelope plasmid (20, 15, and 6 μg, respectively) using Lipofectamine 2000 reagent (Invitrogen), and medium was replaced 7 h after transfection. Two days later, supernatant containing lentiviral particles was collected into fresh 50-ml tubes and maintained on ice. Supernatant was continuously collected twice every 24 h and mixed with supernatant previously harvested (supernatant could be passed through a 0.45-μm filter if necessary to produce purer lentiviral particles). Then we added 40% PEG8000 into supernatant to make a final concentration of 10%. The tubes were incubated on ice overnight. The supernatant/PEG8000 mixture was centrifuged at 4500 rpm at 4 °C for 15 min. Supernatant was aspirated, and tubes were centrifuged again at 4500 rpm at 4 °C for 5 min. We carefully removed the entire supernatant. For a 10-cm dish, precipitant was resuspended by 500 μl of Opti-MEM medium, aliquoted in five 1.5-ml tubes, and stored at −80 °C.

##### Plasmids and siRNAs

DYRK1A expressing vector pCMV-DYRK1A (pDAYRK1A-MycFLAG) and control vector were both purchased from ViGene Bio (Jinan, China). pcDNA4-MycHis_A(+) was purchased from Invitrogen. βTrCP expressing plasmid p4489 FLAG-βTrCP was obtained from Addgene (ID 10865, Cambridge, MA). The kits to produce shβTrCP and control lentiviral particles were purchased from Genechem (Shanghai, China). Ubiquitin-expressing construct was kindly provided by Dr. Weihong Song (University of British Columbia, Vancouver, Canada). Generally, to construct new expressing vectors, the coding sequences were synthesized using polymerase chain reaction or interception from existing plasmids. The coding sequences and the corresponding vectors were both digested with 2–3 restriction endonucleases (New England Biolabs, Beverly, MA). Then the digestion products were separated on 1% agarose gel (Sigma) and extracted with gel extraction kit (Tiangen, Beijing, China). After circling with T4 ligase (New England Biolabs), the ligation products were transferred into *Escherichia coli* DH5α competent bacteria. Ampicillin or kanamycin (Sigma) was used as screening antibiotic.

DYRK1A shRNA-expressing vector was constructed by a former colleague of our laboratory (17). For another DYRK1A-expressing vector pDYRK1A-HA, pDAYRK1A-MycFLAG was digested with BglII and XhoI, and the short fragment was subcloned into pCMV-C-HA (Beyotime Institute of Biotechnology, Haimen, China). For ΔPEST mutant, pDAYRK1A-MycFLAG was digested by BglII, XhoI, and PvuII, and the two short fragments were inserted into pCMV6-entry. The construction strategies for other plasmids were described in Table 1. For siRNAs, sense GUG GAA UUU GUG GAA CAU CTT and antisense GAU GUU CCA CAA AUU CCA CTT for βTrCP were synthesized by GenePharma (Shanghai, China) as described previously (53). To knockdown the expression, siRNAs were transfected into cells using Lipofectamine 2000 reagent (Invitrogen) at 100 pmol/well in a 6-well plate. Forty eight hours later, cells were harvested for analysis.

**TABLE 1:**
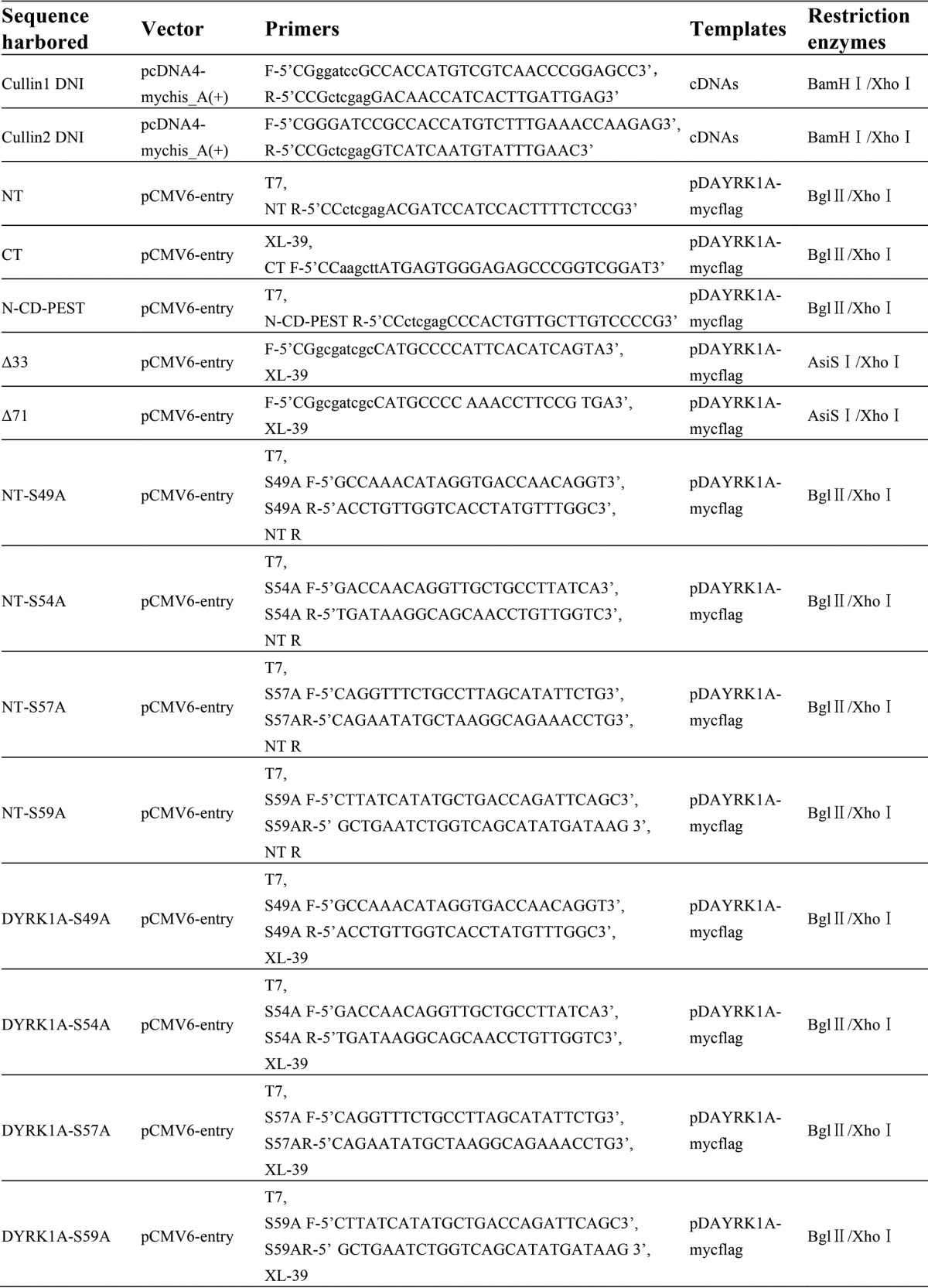
**Cloning primers and vectors**

##### RNA Extraction and Quantitative RT-PCR (qRT-PCR)

Total RNA was purified from cells by TRIzol reagent (Invitrogen), according to the manufacturer's instructions, and cDNA was synthesized from 1 μg of total RNA-utilizing reverse transcription kit (Takara, Dalian, China). For agarose gel electrophoresis analysis, PCR was applied using PCR master mix from BioTeke (Beijing, China). Real time qPCR was performed in an ABI 7900HT Fast Real Time PCR system (Foster City, CA) utilizing SYBR Green PCR Master Mix (Toyobo, Japan) in a 20-μl volume. β-Actin mRNA was used as the internal control. A comparative *Ct* method (2 −ΔΔ*CT*) was used for relative gene expression analysis. Primers were as follows: *ACTB* F, 5′-CACTGTGTTGGCGTACAGGT-3′, and *ACTB* R, 5′-TCATCACCATTGGCAATGAG-3′; β*TrCP* F, 5′-CTGCGGCCTGGCACCAAAGG-3′, and β*TrCP* R, 5′-AGACCGTCCTGGGCCGACTG-3′; and *DYRK1A* F, 5′-GCAATTTCCTGCTCCTCTTG-3′, and *DYRK1A* R, 5′-TTACCCAAGGCTTGTTGTCC-3′.

##### EdU Staining Analysis

One day before staining, cells were plated in a 6-well plate and cultured overnight. The next day, EdU was added to the medium with a final concentration of 10 μm. After 10 h of culture, the cells were trypsinized and stained with Apollo 643 reagent according to the manufacturer's instruction (Ribobio, C00041, China). Stained cells were analyzed on a FACS machine (Beckman, CA) with 20,000 counted.

##### Data Analysis

All the experiments were repeated three times or more. For Western blotting and qRT-PCR, one representative image was shown, and the quantifications were calculated from three or more independent experiments. The values represented the means ± S.E. Student's *t* test and Pearson's correlation test were used for statistical analysis.

## Author Contributions

X. S. designed the experiments. Q. L. performed the experiments. X. S. and Q. L. analyzed the data and wrote the paper. N. L., F. L., P. W., and H. L. helped perform the experiments and prepared reagents. Y. T. and L. C. helped to perform the experiments during the revision.

## Footnotes

2 The abbreviations used are:

DS: Down syndrome
AD: Alzheimer's disease
λ-PPase: λ-protein phosphatase
aa: amino acid
Lac: lactacystin
CHX: cycloheximide
qRT: quantitative RT
REST: RE1 silencing transcription factor
APP: amyloid protein precursor
LB: Lewy body
co-IP: co-immunoprecipitation
F: forward
R: reverse
DN: dominant negative
NT: N terminus
CT: C terminus
βTrCP: β-transducin repeat containing protein
NPC: neural progenitor cell
EdU: 5-ethynyl-2′.
